# Effect of Urban Greening on Incremental PM_2.5_ Concentration During Peak Hours

**DOI:** 10.3389/fpubh.2020.551300

**Published:** 2020-11-16

**Authors:** Shaogu Wang, Shunqi Cheng, Xinhua Qi

**Affiliations:** School of Geography, Fujian Normal University, Fuzhou, China

**Keywords:** green coverage, PM_2.5_, threshold model, kernel-based regularized least squares model, urban maintaining expenditure

## Abstract

In China, severe haze is a major public health concern affecting residents' health and well-being. This study used hourly air quality monitoring data from 285 cities in China to analyze the effect of green coverage (GC) and other economic variables on the incremental PM_2.5_ concentration (ΔPM_2.5_) during peak hours. To detect possible non-linear and interaction effect between predictive variables, a kernel-based regularized least squares (KRLS) model was used for empirical analysis. The results show that there was considerable heterogeneity between cities regarding marginal effect of GC on ΔPM_2.5_, which could potentially be explained by different seasons, latitude, urban maintenance expenditure (UE), real GDP per capita (PG), and population density (PD). Also described in this study, in cities with high UE, the growth of GC, PG, and PD always remain a positive impact on mitigation of haze pollution. This shows that government expenditure on urban maintenance can reduce or mitigate the environmental pollution from economic development. In addition, the influence of other urban elements on air quality had also been analyzed so that different combinations of mitigation policies are proposed for different regions in this study to meet the mitigation targets.

## Introduction

With the rapid urbanization and economic development, urban greening has become an urban livability standard and important symbol of residents' well-being (1). How to achieve a win-win situation for environmental protection and urban development has long been controversial for policymakers (2). Especially for rapidly-developing countries such as China and India, where rapid urbanization and deteriorating air quality is increasingly threatening the well-being of urban residents (3), also increasingly demanded for urban planning, policy, and management.

Over the past decades, numerous studies have highlighted the contribution of urban greening in the achievement of improved air quality, on the basis that pollutants deposit more efficiently onto vegetation (4–6). In a study of broad-scale estimates of PM_2.5_ removal by trees from 10 American cities, substantial health improvements and economic value produced by urban trees have been found (4). In a comparative study in sample cities in China and United States, the authors pointed out that increasing forest coverage of cities through urban greening and afforestation should be a prioritized strategy to mitigate PM_2.5_ pollution, and it was argued, relative to American cities, it was more important for the densely populated and rapidly expanding urban areas in eastern China to increase the intermixing of forest and urban land through polycentric urban development (7).

At a finer spatial scale, a series of methods and tools, such as remote sensing (8–10), field measurements (11–13), Geographical Information Systems (GIS) (10), and landscape analysis software (8), had been used for analyzing urban morphology (building layout, road patterns, land uses, and green space) and its relationship to haze pollution, aims at reducing PM_2.5_ concentrations and minimizing human exposure to particulate matter. A study conducted in the United Kingdom showed that green infrastructures are beneficial but they do not represent a solution to completely remove air pollution from cities, while working on removing street pollution via dispersion proved to be as or even more efficient than deposition technologies (12). In the same article, it was also suggested that areas with leafless period need to consider different reduction in PM_2.5_ caused by seasonal factors like urban greening. In addition, other scholars had also highlighted that pollutant concentration (8, 9, 14) and wind speed (9) should also be considered synthetically since the marginal benefit of urban green coverage on air quality improvement is not always positive under the influence of these factors. In a urban-based panel study, the overall findings support higher density as opposed to lower density urban development in China while higher density does reduce a city's urban park and green space (per capita) (15). The above-mentioned research led us to focus on the necessity of whether urban greening being coordinated with population density, economic growth and other urban elements.

In addition to urban physical elements, there are also studies that attempt to explain the impact of development strategy of cities and policies on air quality. Most empirical studies on this topic have shown that there is a complicated relationship between environmental pollution and the level of socioeconomic development (7, 14, 16, 17). Using panel threshold model, Xiao had revealed the existence of an inverted U-shaped relationship between environmental regulations and PM_2.5_ emissions among 30 OECD countries (16), clearly indicating the importance to develop environmental management and policies in line with the stage of economic development. In contrast to Xiao, Kui argued that haze pollution is a problem derived from the mode of economic development rather than economic development overall, in an empirical study conducted in China, and pointed out that the impact of urbanization varies across regions; while promoting urbanization will be conducive to decreased PM_2.5_ concentrations in Northwest China and Northeast China, it will contribute to increased PM_2.5_ concentrations in other regions (14).

In recent years, spatial analysis or econometric methods have been wildly used in research on influencing factors of urban air quality (18–21), Innovative methods such as multi-scale geographically weighted regression (MGER) (22), semi-parametric global vector autoregressive model (SGVAR) (23) have emerged recently. The spatial effect of haze pollution is becoming an important topic in this field (24, 25).

Previous studies have confirmed the complex correlation between urban greening and haze pollution, but the empirical analysis is still lagging. First, studies have shown that urban air quality is the result of the complex interaction of greening (4, 8, 12, 18), urban form (7, 10, 15), socioeconomic characteristics (11, 14, 19, 20), and regional patterns of development (14, 16), any attempt to statistically assess the correlation between greening coverage and haze pollution will be complicated by a series of confounding factors' variation over time and space, making it difficult to draw general conclusions (7, 24). Distinctive findings in various regions have confused the relationship between greening coverage and air pollution, and the potential of changing air pollution by increasing greening coverage from the perspective of urban planning is still uncertain. Secondly, in the study of horizontal comparison of multiple cities with background PM_2.5_ concentration as the research object, meteorology and activities related to anthropogenic emission are usually the main influencing factors, and few studies directly focus on the effect of greening coverage on air quality. Therefore, more detailed timing data (especially during peak periods) are needed to reflect the improvement of haze pollution by urban greening. Third, studies at different spatial scales have revealed that the effect of green cover on PM_2.5_ concentrations is not a simple linear relationship, which influenced by many other factors such as regional development patterns (14, 16), urban compactness (14, 15), background PM_2.5_ concentrations, vegetation types, wind speed, etc., but the specific proportional relationship between them remains unclear.

This study set out to explore the contribution of green cover and other elements to reduce haze pollution, and further to evaluate the roles of these elements in terms of both singular and interacting behaviors. Those results would be helpful to formulate effective strategies for improving the urban atmosphere environment. To that end, we use ground-level PM_2.5_ data during peak hours in 285 cities in China, which had been undertaken to eliminate regional background concentration. Besides, people were most exposed and vulnerable to haze pollution during peak hours, indicating that the potential health benefits of reducing ambient PM_2.5_ was credible. In addition to adding environmental and urban characteristics as control variable, the model also considers variations in the direction and intensity of each driver in different contexts, such as season, latitude (in consideration of the difference in terrain, climate and variation in vegetation composition caused by 32°N latitude limit), government expenditure (for urban maintenance), etc. Finally, to capture the compounding effects of urban physical parameters on PM_2.5_, the kernel-based regularized least squares (KRLS) model was adopted to consider possible interactions among variables.

As of late, numerous methods were reported to characterize the effect of air quality factors varying along different spatial distribution and conditional distribution such as local linear method (23), geographically weighted regression (GWR) (25), quantile regression (26). The kernel-based regularized least squares (KRLS) algorithms have notable advantages over conventional statistical models. Kernel functions, which provide a measure of similarity between the covariate vectors of two observations as basic functions, complex relationship between dependent variable and independent variable vector, will be translated into a linear combination of these basis functions, thus providing closed-form estimates for the predicted values, variances, and the pointwise partial derivatives that characterize the marginal effects of each independent variable at each data point in the covariate space (27). Therefore, with KRLS analysis, it becomes feasible to capture possible non-linearities, interactions, and heterogeneous effects of factors on mitigation of haze pollution in different cities, consequently, to set corresponding governance measures according to the socioeconomic environment and pollution characteristics in different regions.

## Research Design

### Dependent Variable: PM_2.5_ Data

The ground-level PM_2.5_ data adopted in this study have covered 285 cities in China (1,534 weather stations in total, evenly distributed in the built area), published hourly by China National Environmental Monitoring Center^1^, from 1 June 2017 to 31 May 2018.

Related studies have found that the PM_2.5_ contribution of transportation to average mass concentration can be 25–50% (28–30), other sources also include industrial activities (including electricity generation, industrial fuels) (31, 32), coal burning and biomass combustion for cooking (33), winter heating (34), construction (35), and other specific activities (setting off fireworks & open straw burning) (36, 37). Any attempt to statistically evaluate the strength of association between urban elements and PM_2.5_ pollution will be complicated by a range of confounding factors (7), thus data screening should be undertaken in studies of daily PM_2.5_ concentrations to screen for specific pollution events (11, 13, 38).

**Figure 1:**
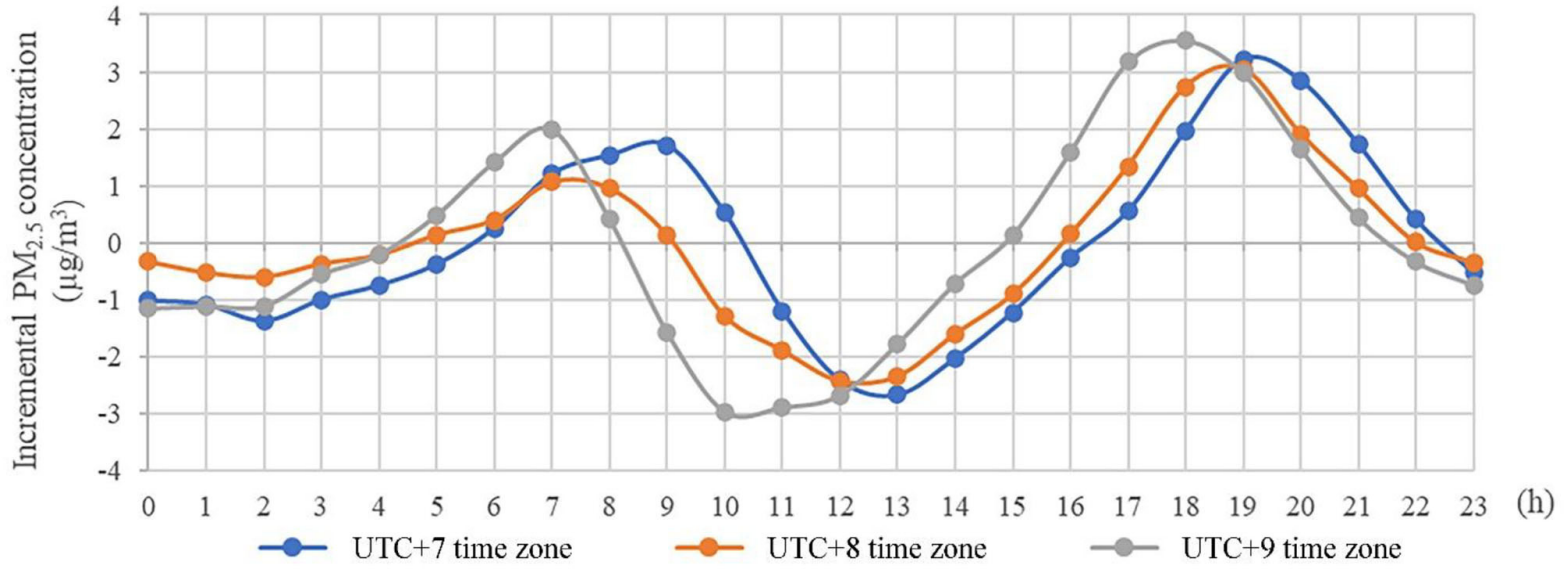
Diurnal variation of PM_2.5_ concentration in different time zones. Karamay and Urumqi, the only two cities in UTC+6 time zone are not considered.

Figure 1 shows the distribution of PM_2.5_ concentration increase per hour from UTC+7 time zone to UTC+9 time zone for a total of 283 cities (Karamay and Urumqi, the only two cities in UTC+6 time zone are not considered) (The detailed average hourly trends of PM_2.5_ concentration in each city are shown in Figure S1). In most cities, PM_2.5_ concentration exhibits a bimodal pattern of changing rhythms, peaking around dawn and dusk, relatively stable at night. Due to the difference in urban morphology (urban size, road conditions, etc.) and time zones, the start time and duration of the increase of particulate matter were different, complex formulas were therefore needed to ensure that the maintenance duration and intensity of the increase in PM_2.5_ concentrations from mobile source are accounted for [considering that the hazard intensity of haze pollution depended on its concentration and exposure time (39–41)]. Then, PM_2.5_ concentration changes from 4:00 to 10:00 a.m. were selected for comparison in this study. These values were converted to μg/m^3^ and expressed herein as Δ*PM*.

(1) $$\begin{array}{r} \Delta PM_{i}=\overset{10}{\underset{t=4}{\sum }}P_{i}^{t}\times I\left(P_{i}^{t}\right) \end{array}$$

(2) $$I(P_{i}^{t})\text{ =}\{\begin{array}{ll} 0 & P_{i}^{t}\leq M\\ 1 & P_{i}^{t}>M \end{array}$$

Where i = (1, ……, N) represents the city number, $P_{i}^{t}$ is the variable quantity of PM_2.5_ concentration per hour (calculated as 0 if the amount of change is negative), I( ) is an indicative function, M is the median of PM_2.5_ concentration per hour in the city i from 4:00 to 10:00 a.m.

The advantages and limitations of such data processing method further explored in the Discussion. The peak period in the morning rather than in the evening was chosen due to, first, commuting activities are relatively single and fixed human activities during the period, which ensure the comparability between regions, while afternoon commutes in parts of China are affected by seasonal changes. Furthermore, PM_2.5_ concentration tends to a source/sink balance at dawn, ensuring a relatively consistent initial state.

### Independent Variables: Socio-Economic Data

Sources of variables used in this study were as follows: China Urban Statistical Yearbook 2018, CEIC China Economic Database, Cathay Pacific Database, National Climatic Data Center^2^. For the missing values in some existing data sets, they are replaced by the corresponding means.

Prior to analysis, all variables were screened by backward elimination statistical procedure, the final model retained five independent variables: (1) Urban green space is a key factor affecting the health and quality of life of urban ecosystem. Local governments in China tend to plant trees rather than build parks to ensure the supply of public goods for urban greening (41). Green space is a kind of non-competitive urban public amenities. To highlight the environmental pressure caused by urban overcrowding, the green coverage per capita (GC) in built-up areas is used to measure the level of urban greening. (2) Within the spectrum of city sizes there are enormous differences in population size, built-up area, and industrial structure. Accordingly, real GDP per capita (PG) is chosen to measure the level of economic development of a region. (3) Increased population density due to urbanization usually leads to increased environmental pressure. Since there are also suggestions in the literature that urban density promotes green travel (reducing gasoline consumption, increasing bicycle use) and thus reduces regional air pollution levels (15, 42), population density (PD) is used replacing urbanization as the main explanatory variable to determine the impact of population density on haze pollution. (4) China's industrial sector consumes far more energy than other sectors and fossil fuel combustion and industrial soot emissions from the secondary industry as well as building dust are important causes of haze pollution (43, 44), thus industrial soot (dust) emissions (IE) is chosen to measure the contribution of industrial production processes to haze pollution. (5) Climatic conditions are key variables affecting the rate of tree deposition, higher air humidity increases PM_2.5_ moisture content, which contributes to the PM_2.5_ settlement in time (5). Therefore, the average annual air humidity of each region is included in the explanatory variables.

China covers many degrees of latitude, with complicated terrain and radical variations in climate, which produces significant variation in north-south vegetation composition. For the final model, we also added a vegetation-type dummy variable N/S in order to control for season-variant fixed effects such that each city was assigned a value of 1 (deciduous vegetation) or 0 (evergreen vegetation) according to whether it was or was not at north of 32°N latitude limit, well-known as the so-called Qinling-Huaihe line (around 32° N in the eastern part of China), which is also the generally accepted boundary line of heating (45).

The primary goal of our study was to understand the effects of greening in particular on mitigation of haze pollution during peak hours. Previous studies have typically examined the impact of government expenditure on air quality from the perspective of pollution control such as energy use and production structure (46), neglected the possibility that urban maintenance expenditure could have a beneficial impact on air quality improvement by expanding the share of greening reward to reduce air pollution. Then, a dummy covariate UE is added to distinguish cities with high-level expenditure (UE assigned as 1) and low-level expenditure (UE assigned as 0) in urban maintenance, which can help determine if government urban maintenance efforts will work best for environmental benefits of greening.

Variable descriptions are in Table 1. The result of backward stepwise regression model is presented in Table 2.

**Table 1:** Data source of variables.

**Variables**	**Definition**	**Mean**	**S.D.**	**Data source**
ΔPM_2.5_	Incremental PM_2.5_ concentration from 4:00 a.m. to 10:00 a.m. (μg/m3)	4.11	3.532	China Environmental Monitoring Station
GC	Green coverage per capita in municipal districts (m^2^)	15.25	13.055	China Urban Statistical Yearbook 2018
PD	Population density in municipal districts (pop/km^2^)	399.33	314.736	CEIC China economic database/Cathay Pacific Database
PG	Real GDP per capita in municipal districts (RMB)	69740.36	35119.019	China Urban Statistical Yearbook 2018
IE	Industrial soot (dust) emissions in municipal districts (tons)	18268.57	22337.091	China Urban Statistical Yearbook 2018
HU	Annual air humidity (%)	68.23	10.751	National Climatic Data Center
UE	The ratio of urban maintenance expenditure to GDP (1 = over 5%, 0 =below 5%)	0.239	-	China Urban Statistical Yearbook 2018
N/S	Whether at north of Qinling-Huaihe line (1 = 32°N North, 0 =32°N South)	0.526	-	CEIC China economic database

**Table 2:** Backward stepwise regression model.

**Variables**	**Coef.**	**Std. Err.**	***t***	***P*>*t***	**VIF**
HU	−1.981	0.333	−5.960	0.000	1.67
PG	−0.345	0.120	−2.890	0.004	1.56
PD	−0.125	0.060	−2.070	0.039	1.43
GC	−0.144	0.078	−1.840	0.067	1.35
IE	0.078	0.046	1.680	0.094	1.16

*Variables were previously logarithmized*.

### Threshold Regression

Threshold model is the basis for developing more complex ones. Therefore, in this study, a threshold model has been applied to explore the non-linear relationship between green coverage and PM_2.5_ concentrations, with refinements and extensions of this model being presented in the following descriptions.

(3) $$\begin{array}{r} y_{i}=\beta_{1}X_{i}\times I\left(q_{i}\leq \gamma \right)+\beta_{2}X_{i}\times I\left(q_{i}>\gamma \right)+\varepsilon_{i} \end{array}$$

*y*_*i*_ is the response variable, *X*_*i*_ is the explanatory variable matrix, β is the coefficient matrix, *q*_*i*_ is the threshold variable, γ is the threshold value to be estimated, and *I*(·) is the indicative function (while *q*_*i*_ ≤ γ, *I*(*q*_*i*_ ≤ γ) = 1, *I*(*q*_*i*_ > γ) = 0, while *q*_*i*_ > γ, *I*(*q*_*i*_ ≤ γ) = 0, *I*(*q*_*i*_ > γ) = 1), ε_*i*_ is the error term.

The arbitrary value of *q*_*i*_ is taken as threshold value for regression of formula (3), and $\hat{\gamma }$ is defined as the estimated threshold value. Therefore, the more approximate the value *q*_*i*_ to the real threshold value $\hat{\gamma }$, the smaller the sum of squares for residuals (SSR) of the model. By carrying out point-by-point regression, it is obtained that when $SSR\left(\hat{\gamma }\right)$ is the minimum, $\hat{\gamma }$ is the estimated threshold value, namely, $\hat{\gamma }=argminSSR\left(\hat{\gamma }\right)$. The important step of threshold regression also includes the determination of the number of threshold value. Generally, Grid Search is used to determine other threshold value that can minimize the sum of squares for residuals. The threshold regression also needs to solve the validity of threshold value. By constructing the maximum likelihood function, the significance and validity tests are carried out. It is assumed that the null hypothesis *H*_0_ of test is θ_1_ = θ_2_ and the alternative hypothesis is θ_1_ ≠ θ_2_. Under the conditions of null hypothesis, the sum of squares for residuals of model regression result is recorded as *S*_0_. Therefore, the statistical magnitude of likelihood test is $LR=\left[S_{0}-S\left(\hat{\gamma }\right)\right]/{\hat{\sigma }}^{2}$. At the same time, with the help of Bootstrap, the asymptotically-efficient interval of *LR* is obtained. The confidence level is set as α. When $LR\leq -2\log\left(1-\sqrt{1-\alpha }\right)$, the null hypothesis is established, which indicates that the model has the threshold effect. In practical application, there may be double threshold or multiple threshold cases, and the double threshold or multiple threshold value can be searched by using similar methods. This study uses several variables to calculate the threshold value for the same sample and selects the optimal variable as the threshold variable according to its significance.

This model referred to the reference model proposed by Hansen (47), the detailed rationale of the model can be found in his paper.

### Kernel Regularized Least Squares (KRLS)

Kernel Regularized Least Squares Model (KRLS) is a machine learning method described in the article by Hainmueller and Hazlett (48), designed to solve regression and classification problems in social science modeling without relying on linear or the additivity hypothesis, allowing interpretation in a manner similar to a generalized linear model, while also allowing the marginal effect of each independent variable in the variable space to be derived. Specific steps are as follows.

#### Kernel Function

Assume a set of observations in the form of (*y*_*i*_, *x*_*i*_), where *i* = 1, …, *N* indexes the observations,*y*_*i*_ ∈ *R* is the outcome of interest, $x_{i}\in R^{D}$, RD is the set of independent variables *x*_*i*_ (*x*_*i*_ can be regarded as a vector composed of D-dimensional variables), using a symmetric, positive definite Gaussian kernel function to measure similarity between the covariate vectors of two observations:

(4) $$\begin{array}{r} k\left(x_{j},x_{i}\right)=\exp\left(-\frac{∥x_{i}-x_{j}{∥}^{2}}{\sigma^{2}}\right) \end{array}$$

Where $∥x_{i}-x_{j}{∥}^{2}$ is the Euclidean distance between the independent variables xi and xj, σ^2^ ∈ *R*^+^ is the bandwidth of kernel function.

Imagine we have some test-point x^*^ at which we would like to evaluate the function value, then the predicted value y^*^ is given by

(5) $$\begin{array}{r} y^{*}=f\left(x^{*}\right)=\overset{N}{\underset{i=1}{\sum }}c_{i}k\left(x^{*},x_{i}\right) \end{array}$$

Where the objective function *f*(x^*^) is regarded as a linear combination of several kernel functions $k\left(x^{*},x_{i}\right)$, and *c*_*i*_ is a weight for each covariate vector.

Since $k\left(x^{*},x_{i}\right)$ is a measure of the similarity between x^*^ and *x*_*i*_, we see that the value of $k\left(x^{*},x_{i}\right)$ will grow larger as we move the test-point x^*^ closer to *x*_*i*_. In other words, the predicted outcome at the test point is given by a weighted sum of how similar the test point is to each observation in the (training) dataset. It is inferred that, similar to the principle of the generalized linear model, we can use a set of kernel functions that describe the similarity of the sample observations to replace the natural measure of the data, that is, for any independent variable x, there should be a weight vector ci(i = 1, …, N), allowing a linear fitting function of the mathematical expected value of the dependent variable to be constructed based on the similarity of the independent variable x to the observed value:

(6) $$\begin{array}{r} y=f\left(x\right)=\overset{N}{\underset{i=1}{\sum }}c_{i}k\left(x,x_{i}\right) \end{array}$$

Applying the Equation (6) to each data set, the model can be rewritten in vector form as:

(7) $$\begin{array}{r} y=Kc=\left[\begin{array}{cccc} k\left(x_{1},x_{1}\right) & \;k\left(x_{1},x_{2}\right) & \;\cdots & \;k\left(x_{1},x_{N}\right)\\ k\left(x_{2},x_{1}\right) & \;\ddots & \; & \;\\ \vdots & \; & \; & \;\\ k\left(x_{N},x_{1}\right) & \; & \; & \;k\left(x_{N},x_{N}\right) \end{array}\right]\left[\begin{array}{c} c_{1}\\ c_{2}\\ c_{N} \end{array}\right] \end{array}$$

In this form, the KRLS model can be viewed as a linear system, the output matrix is a vector of expected values of all dependent variables, with N × N matrix K containing all kernel functions measuring the similarity of observations.

#### Regularization

To find the only approximate solution of Equation (7), with perfect fit being sought by choosing $\overset{\wedge }{c}=K^{-1}y$, it is necessary to control model's fitting bias and complexity at the same time. Therefore, we minimize the model fitting bias and add a penalty term of complexity to construct the objective function:

(8) $$\begin{array}{r} \underset{f\in H}{argmin}\underset{i}{\sum }\left(V\left(y_{i},f\left(x_{i}\right)\right)\right)+\lambda R\left(f\right) \end{array}$$

Where *V*[*y*_*i*_, *f*(*x*_*i*_)] is a loss function that measures the estimated error at each observation, *R*(*f*) is a regular term that measures the complexity of the model, and λ ∈ *R* is a control parameter determining the tradeoff between model fit and complexity. H is the “hypothesis space” formed by all possible functions.

To solve the minimization problem of objective function, the least square method is used to measure the model's variance loss *V*[*y*_*i*_, *f*(*x*_*i*_)], and the Tikhonov regularization method is used to construct complexity penalty *R*(*f*), optimization of objective function in Equation (8) can be expressed as:

(9) $$\underset{i}{\sum }(V(y_{i},f(x_{i})))=\underset{i}{\sum }({\left(f(x_{i})-y_{i}\right)}^{2}={\left(y-Kc\right)}^{T}(y-Kc)$$

(10) $$\begin{array}{r} R\left(f\right)=∥f{∥}_{k}^{2}=\underset{i}{\sum }\underset{j}{\sum }c_{i}c_{j}k\left(x_{i},x_{j}\right)=c^{T}kc \end{array}$$

(11) $$\begin{array}{r} \underset{c\in R^{D}}{argmin}{\left(y-Kc\right)}^{T}\left(y-Kc\right)+\lambda c^{T}Kc \end{array}$$

(12) $$\begin{array}{r} c^{*}={\left(K+\lambda I\right)}^{-1}y \end{array}$$

the kernel bandwidth σ^2^, referring to the method of Hainmueller and Hazlett (48), The default kernel bandwidth σ^2^ is half the average Euclidean distance between the observations after normalization, such that set $\sigma^{2}=D=\frac{1}{2}E\left[∥x_{j}-x_{i}{∥}^{2}\right]$. For the regularization parameter λ, the leave-one-out (LOO) strategy was used to calculates the sum of N observation variances, for any value of λ, and finds the optimal solution of λ by minimizing it (48).

Equation (11) reveals that when the kernel function window width σ^2^ and the regularization parameter λ are fixed, there is c^*^ ∈ *R*^*D*^ such that y^*^ = Kc^*^ is the best linear fit of the conditional expectation function *E*[*y*|*x*, λ, σ]. The objective function is differentiated to obtain the optimal solution *c* of independent weights [Equation (12)]. It should be noted that for each data set in K, the process of generating the independent variable weight c is similar to the linear solution of the mathematical expectation of the dependent variable in the similarity of the sample observations in the fixed function window width, so any independent variable has its corresponding weight c.

#### Partial Derivative Estimation

Assuming that *X* = (*x*^1^, …*x*^*d*^, …*x*^*D*^) is a data set composed of N D-dimensional independent variables, according to Equation (8), any given sample j can be calculated to correspond to the d-dimensional independent variable partial derivative of the objective function:

(13) $$\begin{array}{r} \frac{\overset{\wedge }{\partial }y}{\partial x_{j}^{d}}=\frac{-2}{\sigma^{2}}\underset{i}{\sum }c_{i}\exp\left(-\frac{∥x_{i}-x_{j}{∥}^{2}}{\sigma^{2}}\right)\left(x_{i}^{d}-x_{j}^{d}\right) \end{array}$$

By calculating the point-by-point partial derivative of the d-dimensional independent variable, the average marginal influence of the d-dimensional independent variable on the dependent variable can be obtained

(14) $$\begin{array}{r} E_{N}\left(\frac{\overset{\wedge }{\partial }y}{\partial x_{j}^{d}}\right)=\frac{-2}{\sigma^{2}N}\underset{j}{\sum }\underset{i}{\sum }c_{i}\exp\left(-\frac{∥x_{i}-x_{j}{∥}^{2}}{\sigma^{2}}\right)\left(x_{i}^{d}-x_{j}^{d}\right).\; \end{array}$$

## Results

### Particulate Matter Mass Concentrations and Spatial-Temporal Characteristics

**Figure 2:**
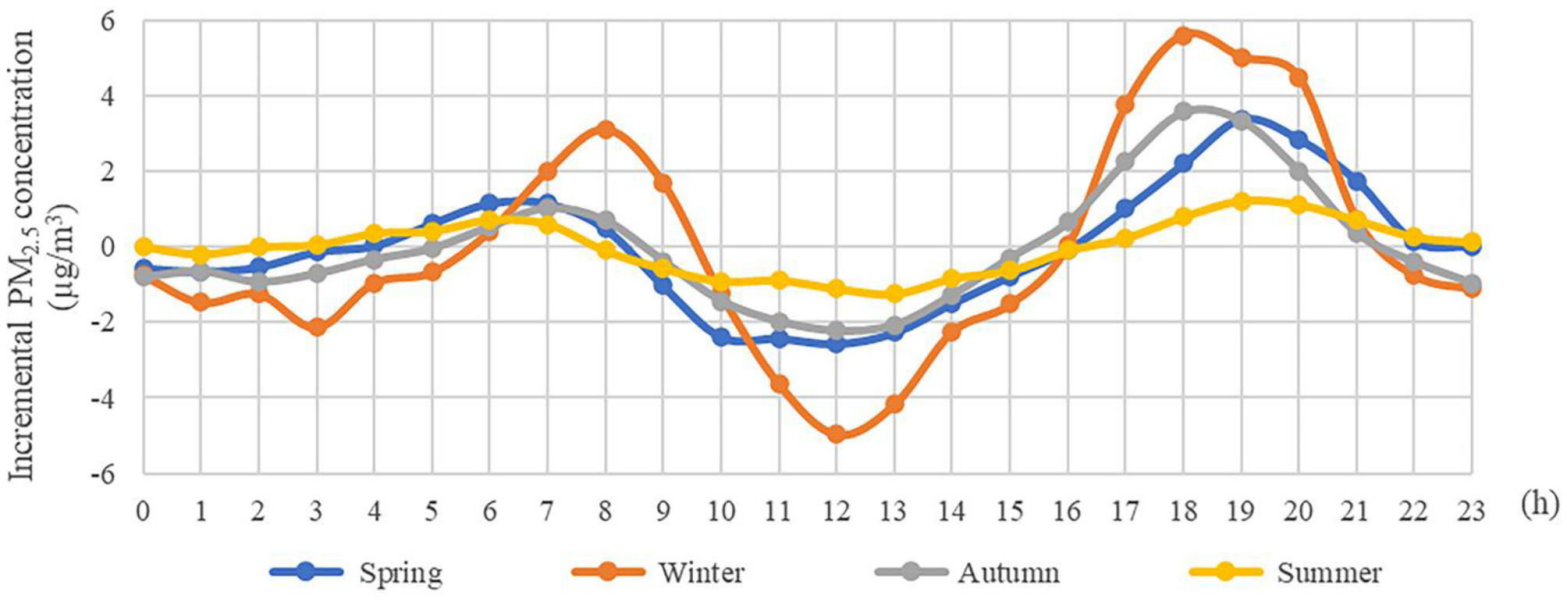
Diurnal variation of PM_2.5_ concentration in different seasons.

Evident seasonal variations of ΔPM_2.5_ concentration increment in 285 cities are illustrated in Figure 2. We found that the amount of growth in PM_2.5_ was significantly higher in winter than leaf-period, which demonstrates the necessity of building different models to capture spatiotemporal trends.

**Figure 3:**
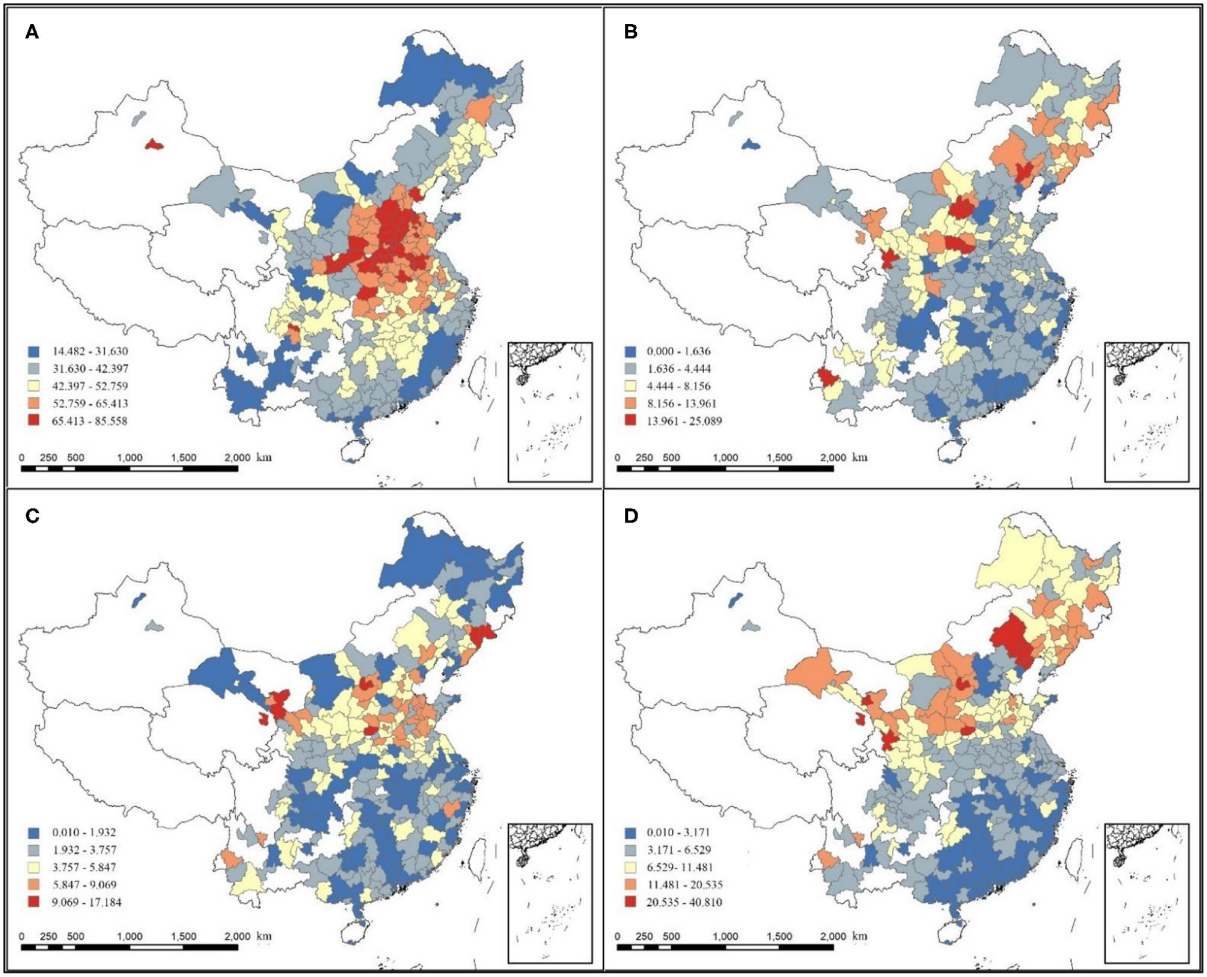
Concentration spatial patterns of: **(A)** Average annual PM_2.5_ concentration; **(B)** Annual incremental PM_2.5_ concentration; **(C)** Incremental PM_2.5_ concentration in summer; **(D)** Incremental PM_2.5_ concentration in winter.

Figure 3 presents the space distribution of PM_2.5_ and ΔPM_2.5_ in all 285 sample cities, which were categorized into five based on Jenks natural breaks. As shown in Figure 3, most of the severe haze pollution was concentrated in plain area of central China. The regions with high ΔPM_2.5_ were distributed mainly in north China and the regions with low ΔPM_2.5_ were distributed mainly in south China, with areas above 32° N experiencing a higher ΔPM_2.5_ in summer than in winter. We can see that a far greater impact on ΔPM_2.5_ is due to changes in season but not spatial spillover effect.

What needs illustration is that, in most cities, the diurnal variations of PM_2.5_ concentrations were largely consistent and showed a bimodal pattern. In summer and winter, no increase in PM_2.5_ concentration was detected in four cities during peak hours (in summer: Zhangjiakou, Changsha; in winter: Baoding, Chaozhou). Among them, Zhangjiakou and Changsha belong to Hebei, one of the provinces with the most severe haze pollution in China, not ruling out the possibility that the nighttime factory emissions wiped out the PM_2.5_ increase that should had occurred in the morning. Since it needs to use the logarithmic form of observed value for model calculation, we used 0.01 (minimum value) instead for cities without PM_2.5_ increase.

### Regression Results of Threshold Model

**Figure 4:**
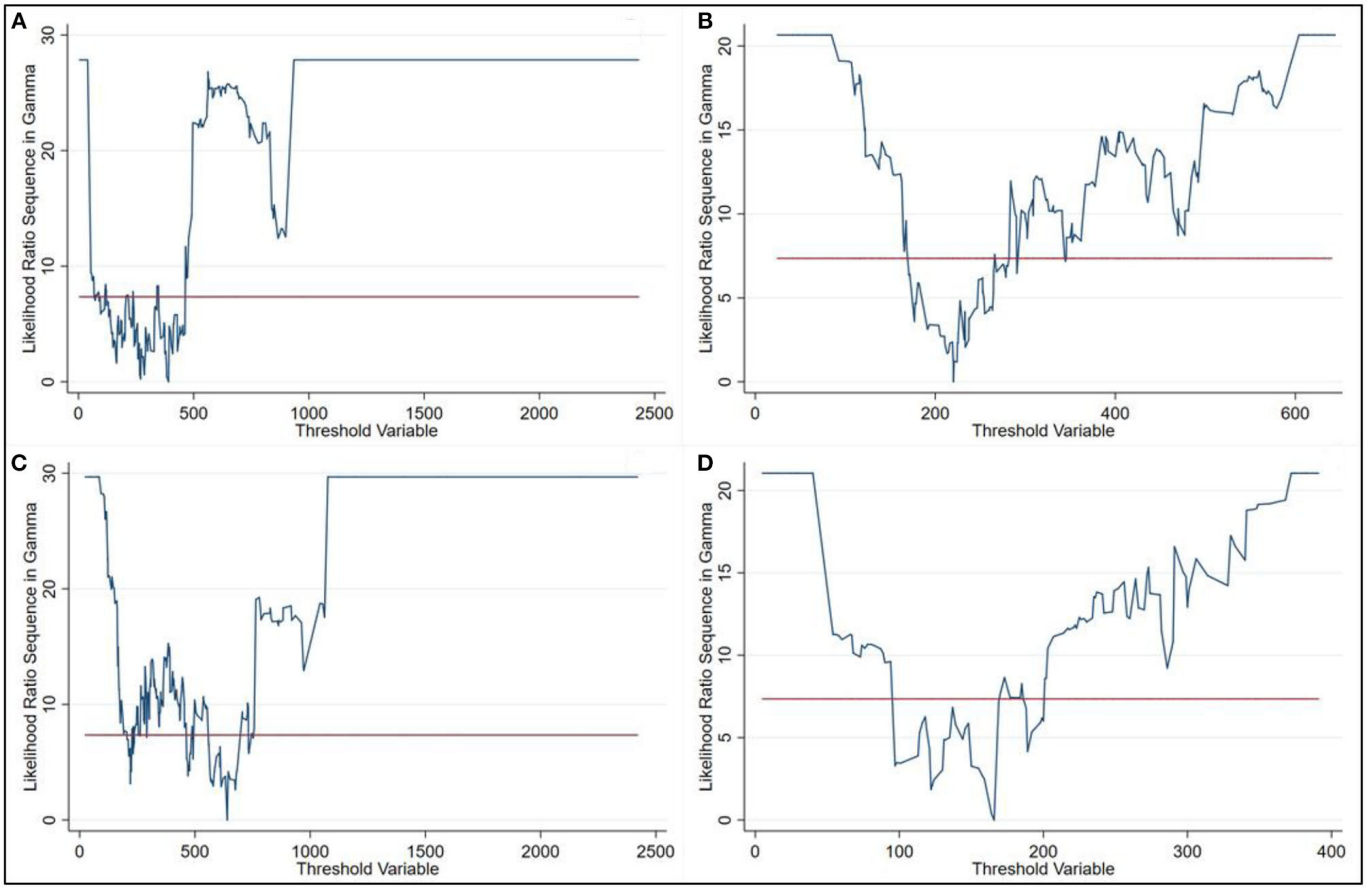
Confidence interval construction in **(A,B)** Urban resident population threshold; **(C,D)** Population density threshold.

A logarithmic version of Hansen's threshold model was estimated using different threshold variables. Table 3 shows the results of the threshold effect tests. Two variables included the threshold effect are significant at the 10% level. We therefore found two threshold effects at the 5% significance level are found in both two variables in further detection. When urban resident population was set as the threshold variable, the threshold values obtained were 220.18 and 640.22. When population density was set as the threshold variable, the threshold values obtained were 165.78 and 391. As the best way to form confidence intervals for threshold is to form “no-rejection region” using the likelihood-ratio (LR) statistic for tests on threshold estimates, we plot the LR statistic (Figure 4) to display the threshold confidence intervals.

**Table 3:** Test of threshold effect.

**Dependent variable**	**Threshold variable**	***P*-values**	**Threshold values**	**95% confidence interval**
ΔPM_2.5_	Urban resident population	0.016	640.22	[574.3,683.064]
	Population density	0.010	391	[97.06,462]
	Proportion of secondary industries	0.184		
	Proportion of tertiary industries	0.384		
	Real GDP per capita	0.564		
	Urban Resident Population ≤ 640.22	0.03	220.18	[168.3,290.69]
	Urban Resident Population > 640.22	0.838		
	Population density ≤ 391	0.044	165.78	[97.06,200]
	Population density > 391	0.690		

**Figure 5:**
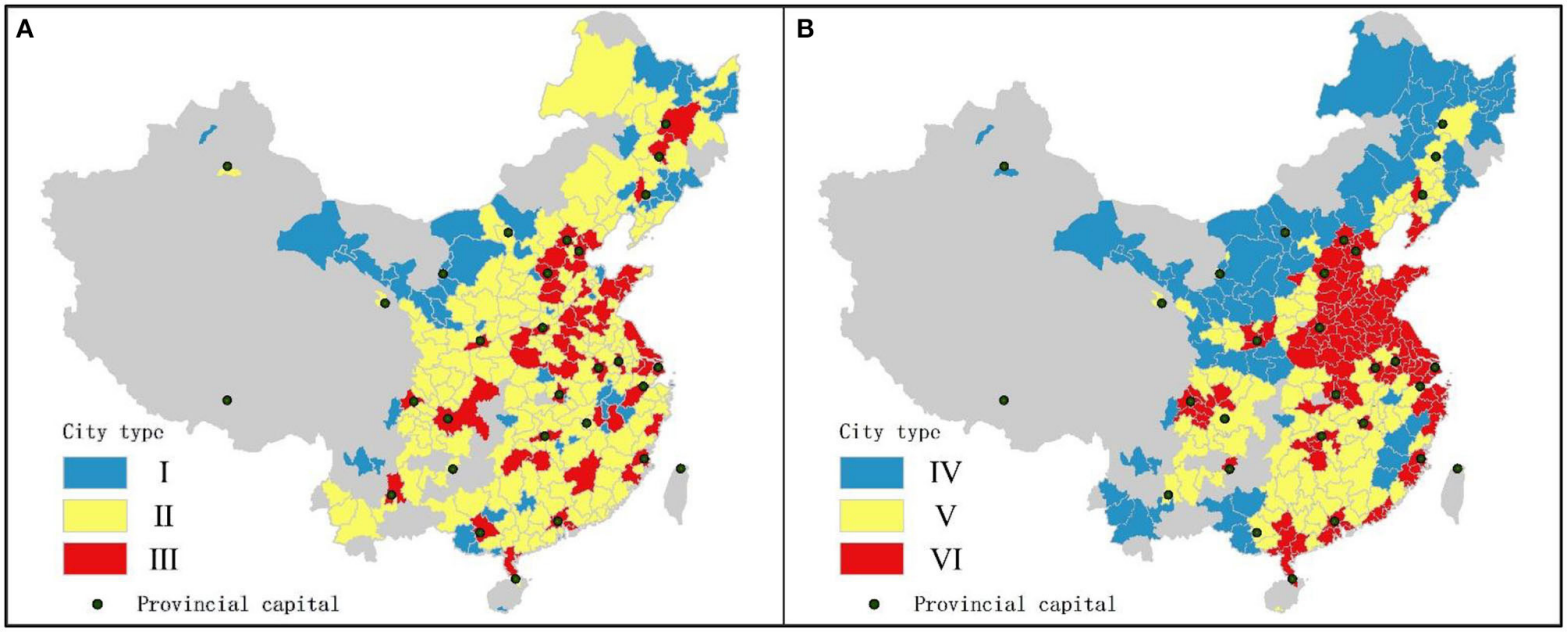
Concentration spatial patterns of cities samples by: **(A)** Urban resident population threshold; **(B)** Population density threshold.

Table 4 presents the effects greening coverage and other factors on ΔPM_2.5_ by using threshold regressions. As the results show, green coverage in cities with low population size had a positive effect on PM_2.5_ at 1% significance level, with a coefficient of −0.344, while no significant correlation was found in the cities of medium and higher population size (Urban resident population >220.18 million). In models with population density as threshold variable, cities with medium population density size was negatively correlated with GC at 1% significance level with a coefficient of −0.443. This means that, for countries with low population (column I) or medium population density size (column V), a 1% increase in green coverage caused a 0.3~0.4% reduction in ΔPM_2.5_ concentrations. Despite the significant negative correlation observed in both column I and column V between GC and ΔPM_2.5_, cities in two columns are non-coplanar in space (Figure 5). This translates into omissions in threshold regression models, a diverse set of non-regular regression models that all depend on specific individual threshold, but a one-threshold model is the basis for developing more complex ones.

**Table 4:** Threshold regression model estimation.

**Dependent variable: ΔPM_2.5_**						
**Threshold**	**I**	**II**	**III**	**IV**	**V**	**VI**
	**Pop < = 220.18**	**220.18 < Pop < = 640.22**	**Pop > 640.22**	**Den < = 165.78**	**165.78 < Den < = 391**	**Den > 391**
GC	−0.344^**^	−0.118	−0.220	−0.095	−0.443^**^	0.125
HU	−0.563	−1.491^*^	−1.441	−1.567^**^	−1.987	−0.571
PD	0.067	−0.142	0.014	0.406^**^	0.048	0.105
IE	0.097	0.053	0.395^*^	−0.104	0.029	0.172^*^
PG	0.029	−0.351^*^	−0.139	−0.056	0.057	−0.666^**^
N/S	0.434	0.354	0.501	0.069	0.591	0.402
R^2^	0.326	0.345	0.308	0.244	0.397	0.217
Obs	60	172	53	68	94	123
**Dependent variable: ΔPM**_**2.5**_**(winter)**						
GC	−0.445^***^	−0.126	−0.148	−0.132	−0.299^*^	0.048
HU	−1.173^*^	−0.122	0.863	−1.484^**^	−2.223	−0.280
PD	0.119	−1.488^*^	−0.136	0.212	0.232	0.100
IE	0.096	0.168^**^	0.455^*^	0.020	0.060	0.245^*^
PG	−0.047	−0.240	0.127	−0.166	−0.009	−0.350
N/S	0.617^*^	0.724^***^	1.453^*^	0.383	0.733^*^	0.961^**^
R^2^	0.552	0.438	0.292	0.361	0.502	0.254
Obs	60	172	53	68	94	123

* *p < 0.05*,

** *p < 0.01*,

*** *p < 0.001*.

Population density was positively correlated with ΔPM_2.5_ concentrations at 1% significance level, suggesting that an increased population density in low density cities could contribute to the increase of air pollution. PG showed a weak negative association with PM_2.5_ concentration in column II, while becoming negative at the 1% significance level in column VI. This indicates that economic growth has obviously positive effect on the mitigation of air pollution in high density cities (population density > 391 pop/km^2^), while the same but weak impact exists in medium-sized cities (220.18 < Urban resident population < = 640.22). Moreover, there was a significant relationship between dummy variable N/S and ΔPM_2.5_.

### Regression Results of KRLS Model

The preceding analyses indicated that the effects of green cover and other factors on ΔPM_2.5_ are not simply linear. Urban resident population threshold is likely evolved from different stages of urban development and economic scale; population density threshold is likely derived from land-sea gradients and latitudinal position. In this context, several sub-objectives were set up and validated by KRLS model to provide reference for urban planners, i.e.,

- To investigate the heterogeneity in the marginal effects of greening on ΔPM_2.5_ at different levels of socio-economic variables.
- What effect does government's strong/weak support for environmental protection have on haze pollution control?
- Can we find trajectories of drivers like EKC theory in relationships between ΔPM_2.5_ and other economic variables?

Traditional OLS regression was conducted along with the KRLS analysis to compare the two statistical approaches (Table 5). Unlike OLS analysis, which presents a constant marginal effect assumption, the KRLS model presents pointwise marginal coefficients for each sample case; therefore, it is possible to observe whether the influence of a given predictor on ΔPM_2.5_ varies with data points. Thus, when the marginal effects are heterogeneous, KRLS results may be informative.

**Table 5:** Results with the OLS and KRLS models.

	**OLS**	**KRLS**				
	**Coef.**	**Avg.**	**SE**	**P25**	**P50**	**P75**
**Variables: ΔPM_2.5_**						
GC	−0.14	−0.129^*^	0.065	−0.262	−0.163	−0.023
HU	−1.065^*^	−1.190^**^	0.367	−1.910	−1.246	−0.510
PD	−0.164^**^	−0.195^***^	0.051	−0.348	−0.217	−0.056
IE	0.0868	0.054	0.038	−0.028	0.049	0.139
PG	−0.307^*^	−0.224^*^	0.091	−0.339	−0.184	−0.051
^*^N/S	0.366^***^	0.327^*^	0.135	0.145	0.285	0.453
R^2^	0.306	0.442				
**Variables: ΔPM**_**2.5**_**(summer)**						
GC	−0.208^*^	−0.157^**^	0.051	−0.265	−0.151	−0.066
HU	−0.955	−0.686^**^	0.207	−0.989	−0.732	−0.393
PD	0.128	0.007	0.042	−0.077	0.001	0.091
IE	0.0926	0.045	0.032	0.001	0.051	0.083
PG	−0.111	−0.059	0.071	−0.186	−0.057	0.058
^*^N/S	0.530^**^	0.412^***^	0.102	0.266	0.442	0.577
R^2^	0.204	0.274				
**Variables: ΔPM**_**2.5**_**(winter)**						
GC	−0.139	−0.092	0.070	−0.185	−0.110	0.027
HU	−1.035	−1.236^**^	0.358	−1.895	−1.423	−0.593
PD	−0.179^*^	−0.188^**^	0.056	−0.310	−0.210	−0.080
IE	0.159^**^	0.111^**^	0.042	0.042	0.104	0.173
PG	−0.223	−0.178	0.099	−0.344	−0.157	−0.023
^*^N/S	0.740^***^	0.550^***^	0.142	0.415	0.566	0.697
R^2^	0.365	0.443				

*Coefficient estimates at the 1st, 2nd, and 3rd quartiles are displayed in Table 5 to show their distribution, and the first column of the KRLS model data indicates the average pointwise marginal effect*.

* *p < 0.05*,

** *p < 0.01*,

*** *p < 0.001*.

Table 5 shows that GC has a positive impact on motivation, the result was significant at the 10 and 5% level in the whole year and winter respectively but not significant in leaf-less period. Considering that deposition of deciduous vegetation on PM_2.5_ in winter may decrease, a further understanding of the interaction between GC and ΔPM_2.5_ is required. The positive correlation between N/S and CR was detected in both statistical models, suggesting that it is reasonable to investigate the effect of urban green cover on air quality in the north and south of Qinling-Huaihe line, which would however require a different isolation protocol.

We also found relationships between economic variable (DE & PG) and ΔPM_2.5_. The significant negative correlation between DE and ΔPM_2.5_ was detected in both statistical models, suggesting that relatively compact urban landscape may be an effective strategy to relieve morning air pollution from human economic activity. PG values also exhibited weakly negative correlation with the ΔPM_2.5_, revealed that economic development is environmentally sound while the mechanism remains to be analyzed. Additionally, a significant positive effect of EM on ΔPM_2.5_ was found during the winter, revealed the need of strict control on pollution emissions in winter.

### Pointwise Marginal Coefficients

The KRLS model was adopted in this study to examine the changes in marginal effects of GC with variation of other variables. This study focused not only on the effects of predictors on haze pollution but also on its heterogenicity varied with latitude and season. In addition, we have also introduced urban maintenance expenditure as control variable, focusing on urban development patterns hidden behind these variables and corresponding mitigation measures.

To evaluate statistically significant interaction effects, additional analyses were conducted by regressing pointwise derivatives of a given independent variable on other predictors, one at a time (49).

**Figure 6:**
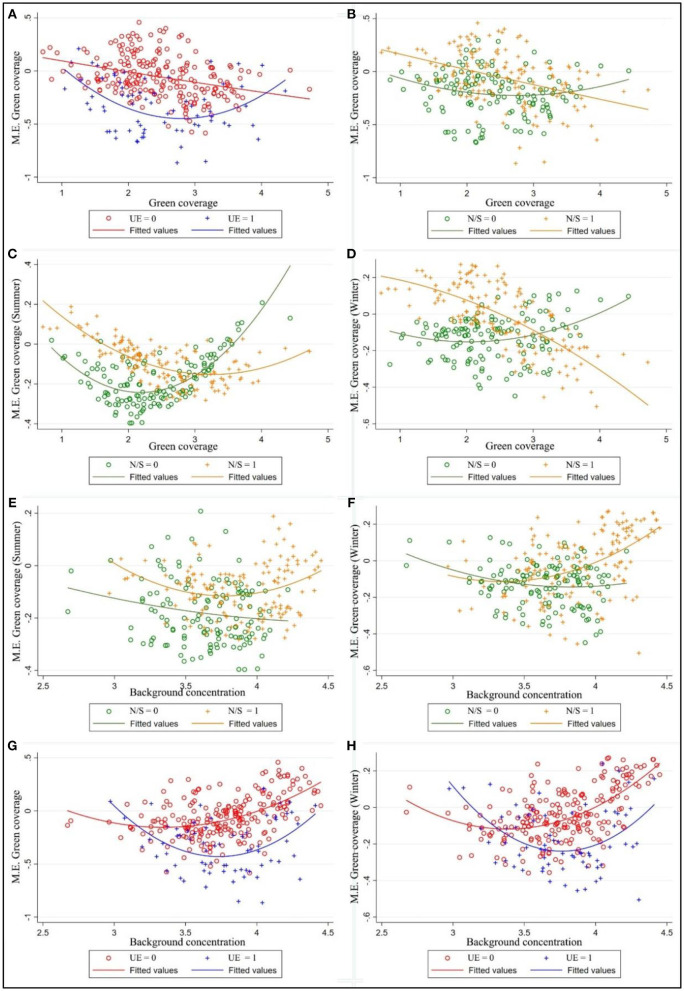
Marginal effects (M.E.) of green coverage varying with values of green coverage **(A–D)** and background PM_2.5_ concentration **(E–H).** Note: The different figure panels represent the heterogeneous marginal effects of GR due to difference in season, dimension, and government environmental expenditure; UE = 1/0 means the ratio of urban maintenance expenditure to GDP greater/less than 5%; N/S = 1/0 means latitude of the city over/below 32°latitude limit.

**Figure 7:**
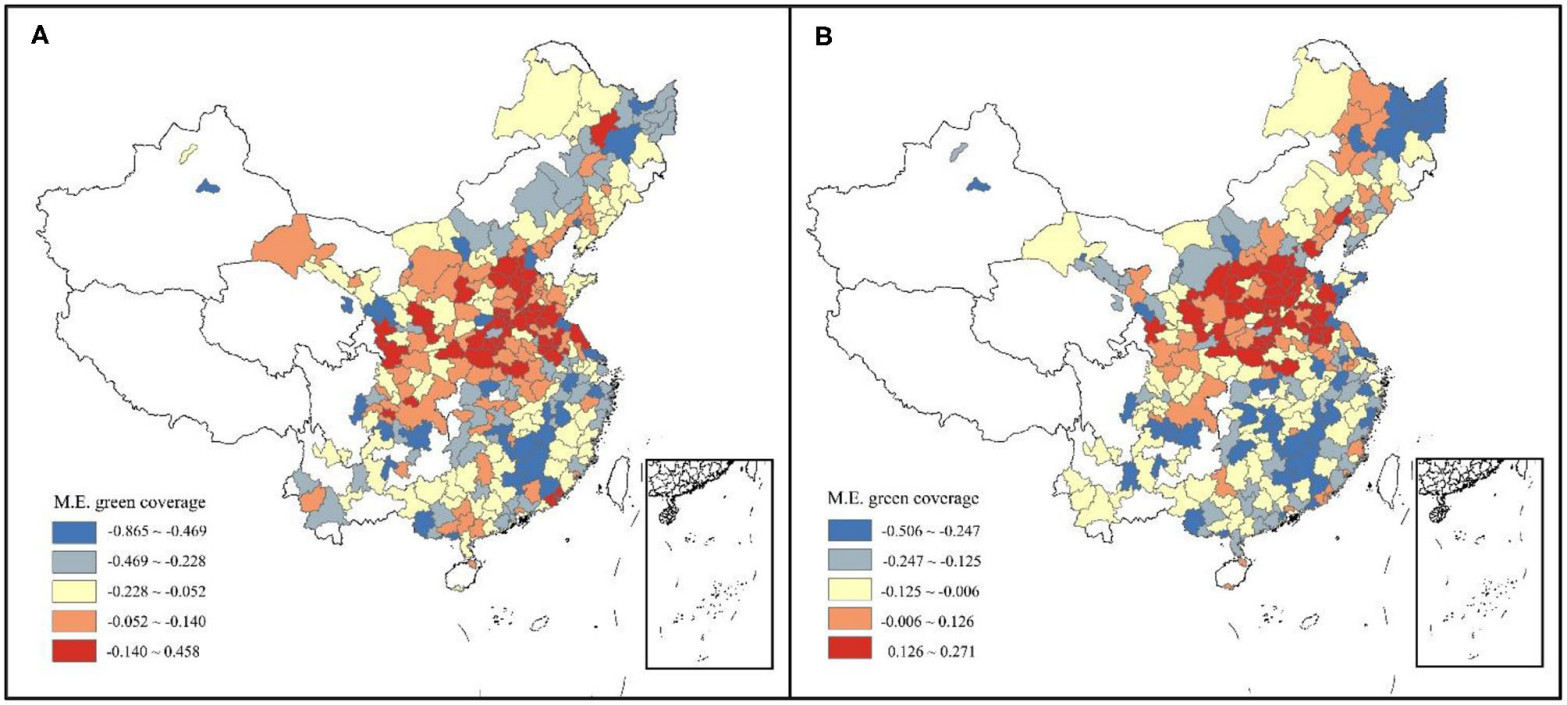
Spatial patterns of marginal effect of green coverage per capita (GC) on: **(A)** Incremental PM_2.5_ concentration; **(B)** Incremental PM_2.5_ concentration in winter.

Figure 6A explored the distribution of the pointwise marginal effects of GC, the negative impacts of GC tend to increase as GC increase while cities with high government environmental investment (above 5% of GDP) having stronger negative driving effect under the same green cover level. This means that government financial support can benefit urban greening. There are no significant North-South differences observed in GC in the Figure 6B. The pointwise marginal effect of GC in summer/winter has been shown in Figures 6C,D considering the large difference of the surface landscape in the north and south regions. It can also be easily inferred from the figures that GC has a constant negative coefficient at all stages in southern citise (latitude < 32°N), but there's a change of sign from positive to negative for that in northern cities. This heterogeneity suggests that other factors interfere with the deposition of particulate matter by vegetation cover. Figures 7A,B further ascertain the spatial distribution of marginal effect of GC, finding that marginal effect of GC, especially during leaf-less period, had high values in areas where the background value of PM_2.5_ was relatively high. Therefore, how the influence of background PM_2.5_ concentration on GC coefficient varies with NS is shown in Figures 6E,F. The increase in the concentration of PM_2.5_ has no significant effect on the coefficient of GC in the southern cities (latitude < 32°N), where levels of pollutants are relatively low. However, for cities located in the north with relatively high background PM_2.5_ concentrations, the increase of GC will lead to the increase of haze concentration, which is especially obvious in winter. But the conclusion is not universal, as is shown in Figures 6G,H, for cities with high government environmental support, the increase of green cover can still effectively reduce haze pollution in high pollution scenarios.

**Figure 8:**
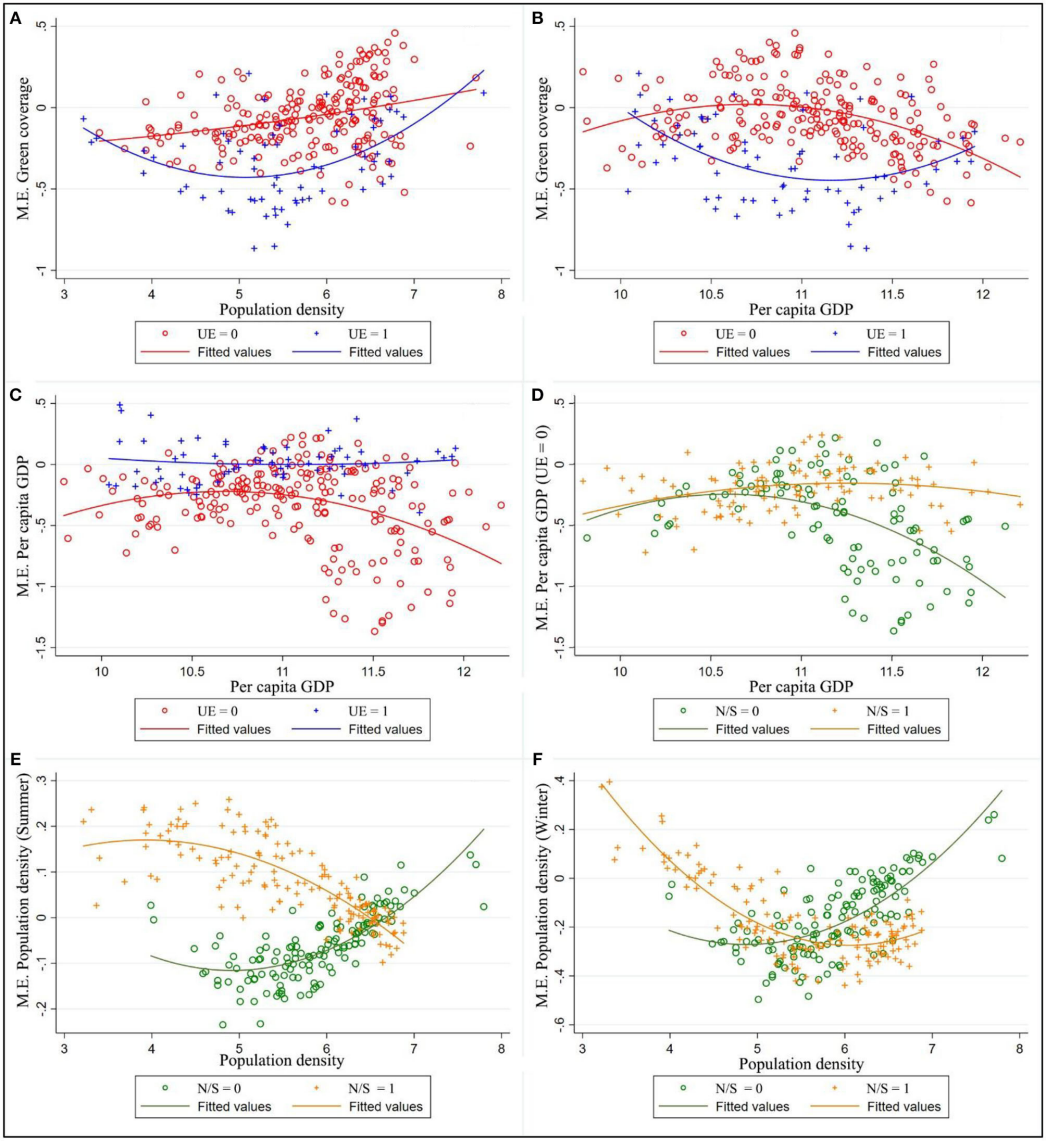
Marginal effects (M.E.) of green coverage **(A,B)**, real GDP per capita **(C,D)** and population density **(E,F)** varying with values of urban elements. The different figure panels represent the heterogeneous marginal effects of GR, PG, PD due to difference in season, dimension, and government environmental expenditure; UE = 1/0 means the ratio of urban maintenance expenditure to GDP greater/less than 5%; N/S = 1/0 means latitude of the city over/below 32°latitude limit.

Figure 8 discussed how other urban elements (population density and economic strength affect their coefficient estimates on ΔPM_2.5_. It can be easily inferred from the Figure 8A that per unit growth of population density could effectively restrain haze pollution in cities with high government environmental expenditure, while in those with low government environmental expenditure, the marginal effects of GC change from negative to positive as population density increases. From the pattern exhibited in Figure 8B, GC appears to function through two pathways in different stages of economic development due to different urban maintenance expenditure (UE). In cities with low UE, Increasing in PG usually leads to higher air pollution during the early stage of economic development. When the economy and income levels reach a certain threshold, the further increase in revenue will improve the environmental quality or reduce the pollution level. In cities with high UE, the marginal effect of GC becoming more noticeable with advancing PG.

The marginal effect of PG and PD were also shown in Figure 8. Figure 8C shows that PG yielded no main effect on dependent variables at high UE; further focus on cities with low UE, as is shown in Figure 8D, PD had no significant effect on ΔPM_2.5_ in northern cities but can effectively improve urban haze pollution in southern, with that effect in developed cities more remarkable. Finally, the bottom graphs Figures 8E,F shows there was heterogeneity of marginal effect of PD differed by season and North-South position. In summer (Figure 8E), increases in PD could decrease ΔPM_2.5_ in southern cities but increased haze pollution in northern cities, the effect fades following the addition of population density such that the marginal effect of PD approached 0 in big cities. In winter (Figure 8F), if exceeded a certain threshold, population density will help mitigate haze pollution.

## Discussion

A significant correlation between GC and PM_2.5_ has been confirmed, based on the above empirical analysis. However, the marginal effect of GC is intricately affected by numerous urban elements, therefore, we use KRLS model to calculate marginal effects of changes in GC at the univariate level.

Urban maintenance expenditure is a new indicator of the importance to environmental protection; it is well-suited to examining ΔPM_2.5_ as an outcome variable since urban maintenance expenditure was observed in this study that has distinguished different urban development patterns, where obvious heterogeneity exists in the impact of greening coverage and other economic variables on haze pollution. Empirical results found that the increase of urban population density and economic intensity will not lead to the decrease of greening effect at the high-level UE phase, where marginal effect from greening is stronger than those with equal GC but low-level UE. In some cities where urban maintenance is relatively neglected, the increase of urban density will lead to the decrease of greening benefit and even the change of coefficient symbol. A similar interaction effects was also found between PG and benefit from GC (Figure 8B), with an increase in PG, the marginal effect of GC displayed a tendency of increasing first and then decreasing at low-level UE. These analyses revealed that urban maintenance expenditure has important implications for air pollution prevention. In China, the state has strict standards for urban green coverage, local government prefers to plant street trees to meet the assessment criteria for its low maintenance costs and no use of construction land. However, “Stresses the construction over the maintenance” may weaken the environmental benefits of urban greening; A large number of street trees to replace the park and grassland landscape will also lead to urban buildings too dense, particulate matter from human activity cannot be dredged. Regrettably, this study has therefore not included urban green space structure as indicators, but we can still find some suggestive support in recent studies. Nowak (5) and Chen (9) declared that vegetation is only a temporary retention site for many atmospheric particles and has its limit in the deposition capability, the leaves will eventually reach the saturation stage and cease to absorb more, as the ambient PM_2.5_ concentration continues to increase (9), such that lead to an increase in PM_2.5_ concentration (12). As a matter of fact, in some areas of the present study (especially in southern cities), it was observed that GC enhanced haze pollution at high-level background PM_2.5_ concentration. Consequently, more consideration should be given to the role of urban greening maintenance and construction (urban forests and parks) in reducing haze pollution.

It was also found that the impacts of PD and GC on ΔPM_2.5_ were different between northern and southern cities, which was very rarely discussed before (14). The purpose of introducing dummy variables N/S was to quantify the unquantifiable variables, as the result demonstrated that such spatial heterogeneity indeed exists. At the same GC level, the mitigation of PM_2.5_ by GC in southern cities was better than that in northern cities. For northern cities, PD had a positive effect on haze growth while significant negative effect may ensue (especially in winter) after reaching the threshold. For northern cities located on the plains with high population density and haze severe pollution, PM_2.5_ concentrations are more dominated by pollution sources (traffic, heating) (38) since extreme growth of PM_2.5_ during peak period could Restrict the settlement of greening as mentioned earlier. For southern cities, increasing population density tends to improve air quality, but in individual cities with particularly high population densities, it also increases haze pollution. One study of 350 cities in the rapidly urbanizing Yangtze River Delta region of China reveals the phenomenon very well: The change in air quality as cities become more spatially sprawled or compact is largely a result of the trade-offs between two counterbalancing effects. On one hand, more geometrically compact and contiguous cities may have reduced vehicle travel distances and toxic air emissions. On the other hand, more spatially sprawled and fragmented cities may have increased intermixing of urban and forest land, and thus may facilitate removal of air pollutant (7). In addition, an extreme situation was also revealed in present study, for northern cities with less GR but higher background PM_2.5_ concentrations, increased GR may exacerbate air pollution during peak hours due to low-level GR having positive effect on ΔPM_2.5_, therefore a vicious circle formed, reflecting the difficulty of haze management in such kind of cities.

The non-linear effect of economic growth on PM_2.5_ emissions has been discussed in numerous studies (14, 16, 19), the conclusion is consistent with the Environmental Kuznets Curve theory. However, this interpretation is more suitable for air pollution from industrial emissions (factory production, urban construction, energy consumption) as government is willing to dramatically increase their budgets under public pressure (16). Our results in this study indicate that urban maintenance expenditure does not depend on levels of economic development, but still performed as well or better than benefit from economic development in mitigation of haze pollution, at least in peak period. On the one hand, the increment of particulate matter during this period may mainly come from the mobile source emissions rather than the industrial emissions [to reduce the harm of industrial emissions to residents, high-pollution enterprises are often located far away from densely populated built areas; due to cheap night electricity prices and pressure from government regulation, most industrial production activities are carried out at night (50)]. On the other hand, commuting, as a rigid demand of urban residents, is less affected by policies. A recent study by Zhang (51) has claimed that the policy measures from industrial sector and residential sector were the major effective control measures, together accounting for 92% of the national abatements in annual PM_2.5_ concentrations, while another measure, strengthening vehicle emission standards, only contribute 2% of that. Therefore, the effectiveness of environmental regulation policies on the increment of particulate matter may seem limited during peak hours.

Based on the refined monitoring data, this study screened out the concentration changes of PM_2.5_ in specific time periods to eliminate the interference of background concentration and spatial spillover effect, ensuring that the samples are comparable. Validation of this practice has been performed in other studies (11, 13, 38). We believe that the use of PM_2.5_ increment instead of original concentration has the following advantages: Firstly, to our knowledge, no cross-sectional study has yet investigated the main emission sources of PM_2.5_ at different times of the day, the present study was designed to begin filling the gap; Secondly, compared with the original concentration, the sudden increase of particulate matter concentration during the peak hours is obviously more related to human activities, mainly commuting traffic, more likely to take feasible measures for improvement. Thirdly, as for peak hours, the higher the concentration and the longer the duration, the greater the harm of haze to health, targeted research can provide positive and effective empirical evidence for decision makers to take measures to reduce the peak value of PM_2.5_ concentration. In addition, since this study used a single-year cross-sectional data, the increment during peak hours, rather than original PM_2.5_ concentration, is instead essential for lowering fixed effects to enable the comparability of data between cities.

As for social science modeling and inference problems, traditional piecewise linear regression usually use indicator variables with regression and classification problems, still relying on linearity or additivity assumptions, this requires that the marginal effect of each covariate is constant in the covariate space. However, this continued marginal effect assumption may be unreliable due to marginal effects being often heterogeneous in levels of other covariates. KRLS draws on machine learning methods designed to solve regression and classification problems without relying on linear or additive assumptions, allows users to tackle regression and classification problems without strong functional form assumptions or a specification search while also permitting more complex interpretation to examine non-linearities, interactions, and heterogeneous effects (27, 49, 50).

As pointed out by reviewers, the present study includes several limitations. First, due to complex interactions between human activities and the atmospheric environment, quantifying the influence of individual factors on PM_2.5_ concentration remains challenging. On the one hand, a few overlooked sources of pollution do exist [e.g., cooking (33, 52), transportation (38), meteorological factors] in this paper. On the other hand, admittedly, emission reduction policy is an important factor affecting PM_2.5_ concentration, since 285 cities involved in this study, it is difficult to quantify policy variables for horizontal comparisons between cities. Factors mentioned above should be included to improve model performance.

Second, the deposition effect of urban green on air quality might be too small to be detected in a single-year cross-sectional regression analysis, as the results of this study confirmed that other factors such as real GDP per capita, urban density, and air humidity have a more significant effect on ΔPM_2.5_ increment. Panel data models can obtain more efficient estimates than cross-sectional data and also reduce the impact of the omitted variable bias because panel data models use more information. It is regrettable that the data used in this paper comes from the China National Environmental Monitoring Center, which has released real-time air quality data since 2015, there will be the issue of independent variable skewness distribution if using a short-term annual data (green coverage in many cities remains unchanged in the short term).

At present, to our knowledge, no cross-sectional study has yet investigated the changes in the daily rhythm of main emission sources of PM_2.5_ in the urban areas, we decided to point to this observation in the discussion, as this provides an interesting starting point for future research. In addition, background PM_2.5_ concentration was an important factor affecting the marginal benefit of greening, it's a pity we have not explored this process specifically, which may inform design of future research studies that further explore these relationships (53).

## Conclusions and Policy Implications

This study investigated the influence of urban greening and other urban elements on incremental concentration of PM_2.5_ during peak hours. Firstly, the temporal (seasonal, diurnal) and spatial variation of incremental PM_2.5_ concentrations in 285 cities in China has been explored. We use the threshold model to make an exploratory analysis on the influence factors of incremental PM_2.5_ concentrations during peak hours (ΔPM_2.5_). For comprehensive results, a KRLS model was used to further explore the non-linearity, interaction, and heterogeneity among parameters. The main findings were as follows:

In addition to green coverage, the estimation results suggest that population density, real GDP per capita, government environmental investment are essential driving factors affecting ΔPM_2.5_. Additionally, the elasticity and significance of each independent variable to the dependent variable may vary across other independent variables level and will also change with season and latitude. To this end, we divide Chinese cities into three categories and adopt different measures to different regions according to local conditions and specific drivers. The results and implications are presented below.

Class I: Southern cities with low government environmental investment. In this kind of city, green cover and economic growth show strong effect on mitigating haze pollution at their respective high level. It was found that excessive population agglomeration can also exacerbate haze pollution. The green coverage per capita needs to be further improved for better air quality. On the one hand, the government should increase the expenditure of greening and air sector, on the other hand, for the densest urban areas, government should reduce the density of economic activities to allocate green resources rationally.

Class II: Northern cities with low government environmental investment. In this kind of city, population density and green coverage per capita were the main influencing factors of haze during the peak period. For cities in the central plains of China, with high background PM_2.5_ concentration, haze pollution was enhanced by green coverage. Contribution of urban density to PM_2.5_ concentration shows positive and negative effects at low and high levels, respectively. That's to say, more intensive development may have reduced vehicle travel distances and toxic air emissions in big cities. Therefore, the control and supervision of pollution sources is crucial for controlling haze pollution. At the early stages of urban development, mitigation interventions related to urban patterns have the greatest potential, reasonable urban planning should be made to reduce environmental stress for follow-up human activity intensive areas (traffic, green Infrastructure, urban Airway). For cities with high population density and smog pollution, a plausible spatial pattern is needed to reduce the burden of commuting, avoiding excessive population concentration, while offsetting the negative environmental impact of economic activities by improving the efficiency of land resource utilization and establishing more stringent policies to limit the discharge of pollutants from production and life.

Class III: Cities with high government environmental investment. Compared with other categories, it's a healthy urban development model that the increase of GC always effectively reduces haze concentration during peak period. In addition, the increase in population density and economic level also expanded the marginal effect of green coverage to reduce air pollution. Altogether, government environmental expenditure was observed to be a powerful means to reduce haze pollution during peak period in every stage of urban development and every region.

## Data Availability Statement

Publicly available datasets were analyzed in this study. This data can be found here: http://data.cnki.net

http://www.gtarsc.com/

http://www.cnemc.cn/sssj/

https://www.ceicdata.com/zh-hans

ftp://ftp.ncdc.noaa.gov/pub/data/noaa/isd-lite/.

## Author Contributions

SW: designed the manuscript, structured and wrote the manuscript, data processed and map designed, indicator calculation, and result analysis. SC: methodology for KRLS model. XQ: funding acquisition, validation, conceptualization, and supervision. All authors contributed to the article and approved the submitted version.

## Conflict of Interest

The authors declare that the research was conducted in the absence of any commercial or financial relationships that could be construed as a potential conflict of interest.

## Footnotes

^1^http://106.37.208.233:20035/

^2^ftp://ftp.ncdc.noaa.gov/pub/data/noaa/isd-lite/
