# Supplementary material for: Effect of Urban Greening on Incremental PM2.5 Concentration During Peak Hours
Source: Front Public Health. 2020 Nov 16;8:551300. doi: 10.3389/fpubh.2020.551300 (PMC7701305; doi:10.3389/fpubh.2020.551300)
Supplement: Supplementary file 1 [file Presentation_1.pdf]

Supplementary Material

Supplementary Material

1 Supplementary Figures and Tables

1.1 Supplementary Figures

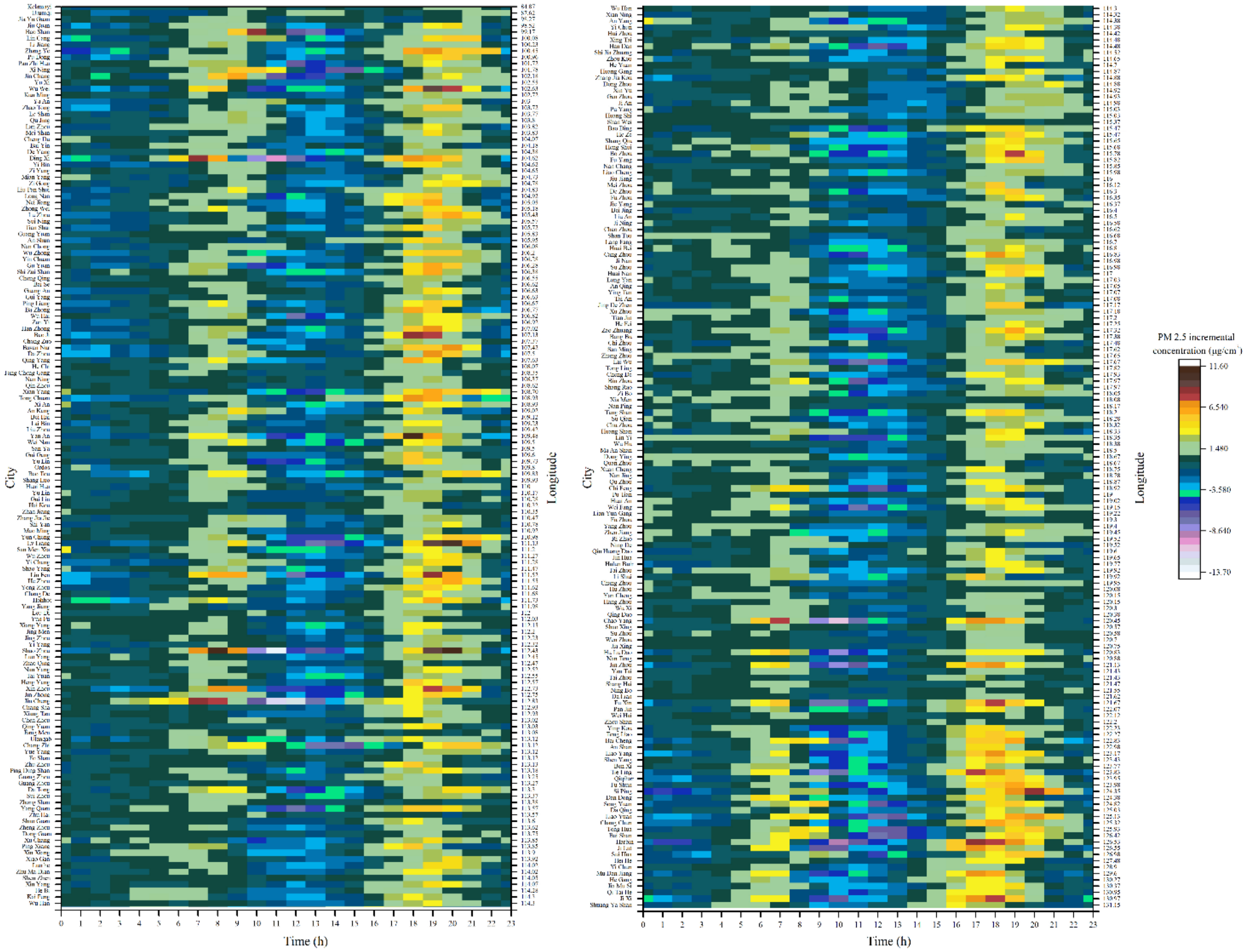

Note: A total of 285 city-specific $PM_{2.5}$ concentrations were averaged by the time of day to observe the diurnal variation from 1 June 2017 to 31 May 2018.

Supplementary Figure 1. Diurnal variation of $PM_{2.5}$ concentration
